# Association Between BoLA-DRB3.2 Polymorphism and Bovine Papillomavirus Infection for Bladder Tumor Risk in Podolica Cattle

**DOI:** 10.3389/fvets.2021.630089

**Published:** 2021-06-09

**Authors:** Maria Longeri, Valeria Russo, Maria Giuseppina Strillacci, Antonella Perillo, Michela Carisetti, Maria Cristina Cozzi, Benedetto Neola, Sante Roperto

**Affiliations:** ^1^Dipartimento di Medicina Veterinaria, Università degli Studi di Milano, Lodi, Italy; ^2^Dipartimento di Medicina Veterinaria e delle Produzioni Animali, Università degli Studi di Napoli Federico II, Naples, Italy; ^3^Dipartimento di Medicina Veterinaria, Università degli Studi di Bari, Bari, Italy; ^4^Istituto Zooprofilattico Sperimentale del Mezzogiorno, Naples, Italy

**Keywords:** bovine papilloma virus type 2, DRB3 exon 2 (DRB3.2), major histocompatibility complex class II (MHC II), bovine leukocyte antigen (BoLA), E5 oncoprotein

## Abstract

Blood samples from 260 unrelated cattle (132 animals affected by papillomavirus-associated bladder tumors and 128 healthy) were genotyped using the classic polymerase chain reaction/restriction fragment length polymorphism method to screen MHC class II bovine leukocyte antigen-DRB3. 2 polymorphism. The DRB3^*^22 allele was significantly (*p* ≤ 0.01) detected in healthy cattle, thus appearing to have a negative association (protective effect) with virus infection of the urinary bladder known to represent a bladder tumor risk for cattle living free at pasture. Considering the two sequence alleles identified in animals carrying DRB3^*^22, DRB3^*^011:01 allele from samples of animals harboring the unexpressed bovine papillomaviruses (BPV)-2 *E5* gene was characterized by amino acid residues believed to have a protective effect against BPV infection such as arginine at position 71 (R^71^) in pocket 4, histidine at position 11 (H^11^) in pocket 6, and both glutamine at position 9 (Q^9^) and serine at position 57 (S^57^) in pocket 9 of the antigen-binding groove. The DRB3^*^011:02v allele from affected animals was characterized by amino acids believed to be susceptibility residues such as lysine (K^71^), tyrosine (Y^11^), glutamic acid (E^9^), and aspartic acid (D^57^) in these pockets. These results suggest that animals harboring the DRB3^*^011:01 allele may have a lower risk of BPV infection and, consequently, a reduced risk of bladder tumors.

## Introduction

Papillomaviruses (PVs) are a heterogeneous group of small, non-enveloped, double-stranded DNA viruses distributed globally. They infect the mucosal and cutaneous epithelia of vertebrates, resulting in benign and malignant lesions of the skin and mucosa (1).

Bovine papillomaviruses (BPVs) comprise 29 members grouped into five genera [http://pave.niaid.nih.gov/, (2, 3)]. BPV-1,−2,−13, and−14, considered to be highly pathogenic (4), belong to *Deltapapillomavirus* genus (δPV).

Bovine δPVs are the only BPVs known to be characterized by natural cross-species transmission and infection (1), including a recent congenital infection that resulted in oral fibropapillomatosis of newborn lambs (5). Bovine δPVs play a central role in bovine and bubaline bladder carcinogenesis (6, 7). Indeed urinary bladder tumors are common in adult cattle and water buffaloes reared fully or partially on hilly/mountain pasturelands rich in bracken fern (*Pteridium* spp.), as the urinary bladder of herbivores is the specific target for bracken genotoxins such as ptaquiloside (PT) (7–10). Indeed PT is responsible for the alkylation of adenine of codon 61 of H-ras gene, resulting in a glutamine 61 substitution that is essential for guanosine triphosphate (GTP) hydrolysis (11). Glutamine substitution at position 61 impairs the intrinsic GTPase activity. Therefore, active GTP-bound conformations (Ras-GTP) accumulate in cells, thereby causing abnormal cell proliferation and differentiation (12).

Bovine δPVs exhibit their transforming activity through the E5 protein, which is the most highly conserved and the smallest known naturally occurring oncoprotein (13). Numerous molecular pathways by which E5 oncoprotein is responsible for cellular transformation of naturally occurring urothelial tumors have been identified. It binds to the transmembrane domain of the platelet-derived growth factor β receptor (PDGFβR), causing dimerization and activation of the receptor (7, 14). E5 has been shown to be involved in bladder carcinogenesis *via* PDGFβR and calpain3 activation pathways (7, 15). In addition, E5 binds to the D subunit of the V_1_-ATPase proton pump (16).

It has been suggested that PT and BPV-2 may act synergistically. PT is an initiation factor responsible for bladder cell hyperplasia, and BPV-2 appears to be a promotion factor (6).

The bovine leukocyte antigen (BoLA) system is the major histocompatibility complex (MHC) of cattle. The MHC genes, mapped to bovine autosome 23, are highly polymorphic and play key roles in immune susceptibility and resistance to pathogens (17). The class II genes encode proteins that present processed antigens to helper T cells bearing the CD4^+^ marker differentiation, thus playing an important role in controlling viral diseases (18). A number of studies have reported the association of one or more of the BoLA-DRB3.2 alleles with susceptibility/resistance to some infectious diseases in cattle such as dermatophilosis and bacterial mastitis (19). Furthermore, it has been shown that MHC genes play a crucial role to foot and mouth disease virus infection (20). Moreover, some MHC class II gene polymorphisms have been associated with resistance and susceptibility to bovine leukemia virus infection (21). In particular, some associations between the MHC genes and human papillomavirus (HPV) and BPV infections have been suggested (22, 23). These associations influence both susceptibility to and regression of PV infections. It has been shown that MHC class II plays an important role in cervical cancer caused by HPV-16 and HPV-18 (24, 25) as well as in horse sarcoids associated with BPV-1 and BPV-2 (26). In PV infections, T cell response is believed to be critical for virus clearance (27).

The bovine MHC comprises three DRB class II copies, with the BoLA-DRB3 gene being the most highly expressed, functional, and polymorphic. DRB3 polymorphism is mainly concentrated in the second exon, which encodes the variable portion of the peptide-binding groove (18). It has been suggested that polymorphisms influence both the magnitude and epitope specificity of antigen-specific T cell responses to infectious diseases. In this sense, the BoLA-DRB3 gene is associated with differences in susceptibility to infectious diseases (17). Antigens are specifically bound *via* interactions of amino acid residues at specific positions of pockets (P) 1, 4, 6, 7, and 9 of the groove. Every MHC class II allele has a narrow or wider specific affinity of binding for a given peptide depending on its different residues in these five pockets (25).

The present study aimed to investigate the potential risk association between BoLA-DRB3 exon two polymorphism and BPV infection of the urinary bladder, which plays a central role in bladder carcinogenesis in Podolica cattle.

## Materials and Methods

### Ethics Statement

We did not perform any animal experiments, and no ethics approval was required.

### Animals and Blood Samples

In this study, we selected and examined 260 blood samples from unrelated cattle that we collected from public slaughterhouses in southern Italy over a span of several years. The samples were from 132 animals suffering from a clinical syndrome called “chronic enzootic hematuria” caused by naturally occurring papillomavirus-associated bladder tumors (“affected”) and from 128 apparently healthy cattle without any bladder lesions. E5 oncoprotein encoded by an early viral gene was found only in affected cattle; on the contrary, all healthy cattle harbor papillomavirus DNA only without any expression. All the animals were slaughtered according to the owners' decisions and after veterinary investigations were carried out as ante-mortem examination in accordance with the national legislation. In both affected and healthy animals, we carried out a post-mortem examination with the permission of the official veterinarian to harvest tissue samples. To prevent possible cross-contaminations, during sampling, both neoplastic and healthy bladder tissues were immediately divided into several parts. Some parts were frozen in liquid nitrogen and stored at −80°C for subsequent molecular biological analysis. The remaining parts were fixed in 10% buffered formalin for microscopic investigations. Blood samples were obtained during slaughtering procedures in sterile containers and immediately stored in EDTA-containing tubes for each sampled animal. They were kept at a refrigerated temperature up to labs. The clinically normal cattle were from the same affected cattle herds located in hilly/mountainous areas of several regions of southern Italy. The affected group was composed of 4–18-year-old cattle; the clinically normal animal group comprises 2–16-year-old cattle. Both groups shared bracken fern-infested lands. All these as big as hectare scattered rangelands, including greenwood floor vegetation, contained an overabundance of bracken fern (*Pteridium aquilinum)*, the most prevalent variety of fern in Southern Italy. Podolica cattle usually graze *ad libitum* on these lands covered with fern fronds that represent the most important source of food from May to August when the plant increases in density. There are no significant differences in bracken fern exposure among all animals living in these conditions.

Methods to collect samples and detect the presence and expression of BPV-2 in healthy and pathological bladders have been described in detail elsewhere (7, 10, 15, 28–31).

### BoLA-DRB3.2 Genotyping and Antigen-Binding Groove Analysis

Genomic DNA was isolated from 200 μl of blood collected in EDTA-containing anticoagulant tubes using the DNeasy® Blood & Tissue Kit (Qiagen^TM^, Wilmington, DE, USA) according to the manufacturer's instructions. BoLA-DRB3 exon two was genotyped according to (32). In short, semi-nested PCRs with primers HL030-5′ATCCTCTCTCTGCAGCACATTTCC; HL031-5′TTTAAATTCGCGCTCACCTCGCCGCT (first round) and HL30 and HL032-5′TCGCCGCTGCACAGTGAAACTCTC (second round) were prepared, and specific amplicons were digested with RsaI, BstYI, and HaeIII run on acrylamide gel. The polymerase chain reaction (PCR)/restriction fragment length polymorphism (RFLP) allelic variants were identified with the combined reading of the three restriction patterns (according to the International Society of Animal Genetics BoLA workshops nomenclature). A subset of 19 samples showing PCR/RFLP allele^*^22 was cloned using the pMOS blunt-ended PCR cloning kit (GE Healthcare, USA). Clone inserts Sanger-sequenced both strands using the ABI PRISM BigDye Terminator Cycle Sequencing Ready Reaction Kit (Thermo Fisher, Scientific, Waltham, MA, USA) to determine the sequence allele because this restriction pattern may include more alleles. The nucleotide sequences were multiple-aligned and translated using ClustalW implemented in BioEdit. Amino acid sequences were aligned to all the BoLA-DRB3 protein sequences downloaded from the (33) using the BLAST tool in NCBI. Amino acid residues forming a pocket of the antigen-binding groove in the BoLA-DR were determined by analogy with HLA-DR. In amino acid motif analysis, classification by charge was made by grouping the amino acids into three categories [positive (R, H, and K), negative (D and E), and uncharged (A, N, C, Q, G, I, L, M, F, P, S, T, W, Y, and V)] and by volume and polarity [one for special (C), two for neutral and small (A, G, P, S, and T), three for polar and relatively small (N, D, Q, and E), four for polar and relatively large (R, H, and K), five for non-polar and relatively small (I, L, M, and V), and six for non-polar and relatively large (F, W, and Y)]. Pocket and residue numbers were reported according to (34); amino acid charge, volume, and polarity were reported according to (35).

### Statistical Analysis

PCR/RFLP allele frequencies and residue motifs were calculated by GENEPOP 3.1d and compared using 2 × 2 contingency tables. The chi-square test (using Yate's continuity correction for the estimation of probability in small samples), *p*-value, odds ratio (OR), and confidence intervals (CI) were calculated by proc GENMOD (© 2006 SAS Institute Inc., Cary, NC, USA) and classical formulae. Fisher's exact test was employed to evaluate the difference in the frequency of amino acid residues in affected and healthy animals. For cases showing a statistically significant difference in frequency between affected and healthy animals, relative risk (RR) was calculated using the following equation: Pd (1 – Pc)/(1 – Pd) Pc, where Pd and Pc are frequencies of the amino acid residues, presumed to be the risk factor for virus infection in the affected (Pd) and healthy (Pc) cattle (34, 36).

## Results

### Virological Findings

For this study, we selected 260 unrelated cattle, 132 of which were affected by BPV-2-associated bladder tumors and 128 were without any bladder lesions (healthy). We have been studying this group for many years in an ongoing research project examining the relationship between BPV infection and bladder cancerogenesis. From each cattle, we have been routinely collecting bladder and blood samples. In all tumor cattle, BPV-2 presence was detected by specific primers, amplifying and sequencing several BPV-2 E5 DNA fragments according to the BPV-2 sequence deposited in GenBank (accession number: M20219.1). Furthermore, in the affected cattle, BPV-2 expression was shown by detecting E5 oncoprotein through both reverse transcription (RT)-PCR showing its transcripts and by Western blot both in bladder tumor and blood samples. In bladder and blood samples from healthy cattle, BPV-2 E5 DNA only was detected by PCR since neither BPV-2 E5 oncoprotein expression by Western blot nor BPV-2 E5 transcripts by RT-PC has ever been shown. All the molecular results were reported elsewhere (7, 10, 15, 29–31). However, in Supplementary Table 1, data about BPV-2 are summarized.

### BoLA Alleles

Overall, 37 PCR/RFLP alleles were identified from 260 animals (Supplementary Table 2), indicating a high variability within the Podolica breed. Eight, namely, DRB3^*^01, ^*^07, ^*^08, ^*^10, ^*^11, ^*^22, ^*^27, and ^*^28, showed an allele frequency higher than 5% and, therefore, were statistically evaluated (Table 1).

**Table 1:** BoLA DRB3.2 PCR/RFLP alleles with a higher frequency than 5% in 260 animals.

PCR/RFLP DRB3.2* allele	**Allele n**.	**Tot Freq**	**Freq in affected**	**Freq in healthy**	**Chi-squared (Yates' correction)**	***p*-value**	**Odds Ratio (95% C.I.)**
1	63	0.12	0.13	0.12	0.02	n.s.	1.08 (0.63–1.82)
7	38	0.07	0.05	0.09	2.61	n.s.	0.54 (0.27–1.07)
8	30	0.06	0.05	0.06	0.03	n.s.	0.88 (0.41–1.83)
10	33	0.06	0.08	0.05	1.82	n.s.	1.76 (0.84–3.65)
11	33	0.06	0.05	0.07	0.66	n.s.	0.7 (0.34–1.42)
22	46	0.09	0.06	0.12	5.89	*p* ≤ 0.01	0.44 (0.23–0.83)
27	34	0.07	0.09	0.04	3.46	n.s.	2.13 (1.01–4.45)
28	47	0.09	0.09	0.09	0.01	n.s.	1.01 (0.55–1.84)

*For each allele, Table 1 reports numerosity and total frequency; allele frequency in the affected and healthy groups; Chi-squared with Yates' correction, and p-value (n.s.: not significant); odds ratio and 95% confidence interval*.

Only one allele showed a high significance (*p* ≤ 0.01), namely, DRB3^*^22, which was found to be prevalent in healthy cattle harboring BPV-2 E5 DNA only without any bladder lesions, which may suggest a negative association with BPV-2 infection, in comparison with the affected cattle in which the BPV-2 *E5* gene was expressed. Since the ^*^22 restriction pattern may include more alleles, to understand whether bladder tumor risk in animals with the BoLA DRB3.2^*^22 allele could be related to differences in the residues of the antigen-binding groove, a subset of ^*^22-carrying samples was cloned and sequenced. Two sequence alleles resulted: BoLA03116|DRB3^*^011:01 and a variant of BoLA09801|BoLA-DRB3^*^011:02 according to the IPD-MHC website.

The variant DRB3^*^011:02v differed from DRB3^*^011:02 only for the substitution of serine (S) with aspartic acid (D) at position 57. Aligning the allele sequences and comparing healthy and affected groups, differences were reported at amino acid residues in three pockets, namely, 4, 6, and 9. An additional residue was different at position 45 in a non-pocket-forming position (Supplementary Table 3).

In particular, arginine (R) was the amino acid residue at position 71 (Arg^71^) in P4 of the BoLA-DRB3^*^011:01 allele of most healthy animals (six of nine, that is 67%—three of which were homozygous), whereas lysine (K) was the prevailing amino acid residue in this position (Lys^71^) in the affected animals carrying BoLA-DRB3^*^011:02v (eight out of 10, which is 80%). Furthermore, with the same percentages, respectively, and consistently due to the linkage between the residues within the alleles (Supplementary Table 3), a large majority of healthy animals with the BoLA-DRB3^*^011:01 allele in the P6 had histidine (H) as amino acid residue at position 11 (His^11^). Tyrosine (Y) was the amino acid residue seen in most affected animals with the BoLA-DRB3^*^011:02v allele at the same position (Tyr^11^). In addition, in the P9 of healthy animals with the BoLA-DRB3^*^011:01 allele, glutamine (Q) was the most prevalent amino acid residue at position 9 (Gln^9^); on the contrary, glutamic acid (E) was the prevalent residue in the BoLA-DRB3^*^011:02v allele of the affected animals (Glu^9^). Serine (S) was the most prevalent residue at position 57 in the P9 of healthy animals with the BoLA-DRB3^*^011:01 (Ser^57^). In affected animals with the BoLA-DRB3^*^011:02v, aspartic acid (D) was the prevalent amino acid residue in this position (Asp^57^). Finally, in the not-pocket-forming position 45, Asp^45^ was the residue mostly found in healthy animals. In affected animals, glycine (G) was found as the prevalent residue in this position (Gly^45^). The amino acid residues and their chemico-physical property differences between healthy and affected animals, as well as BPV infection status, are summarized in Table 2.

**Table 2:** DRB3.2*22 allele and bovine papillomavirus (BPV) infection status.

**allele**		**Pocket 4 Residue 71**	**Pocket 6 Residue 11**	**Pocket 9 Residue 9**	**Pocket 9 Residue 57**	**No Pocket Residue 45**	**BPV infection: prevalent status**
BoLA-DRB3*011:01	AA	R	H	Q	S	D	BPV E5 DNA only: No virus expression
	Charge Volume Polarity	Positive Relatively Large Polar	Positive Relatively Large Polar	Uncharged Relatively Small Polar	Uncharged Small Neutral	Negative Relatively Small Polar	
BoLA-DRB3*011:02v	AA	K	Y	E	D	G	BPV E5 transcripts and oncoprotein: Virus expression
	Charge Volume Polarity	Positive Relatively Large Polar	Uncharged Relatively Large Nonpolar	Negative Relatively Small Polar	Negative Relatively Small Polar	Uncharged Small Neutral	

Differences in amino acid residues between BoLA-DRB3*011:01 (prevalent in cattle harboring BPV DNA only) and BoLA-DRB3*011:02v (prevalent in animals with BPV expression). Pocket and residue numbers are in accordance with Sharif et al. (34), amino acid charge, volume and polarity with (35).

Fisher's exact test showed a *p*-value of 0.02, thus being of statistical significance, and RR, calculated through amino acid residues, was 14.2 in the affected cattle with the BoLA-DRB3^*^011:02v, which indicated a strong positive association with BPV infection; in healthy cattle with the BoLA-DRB3^*^011:01, RR was 0.07, which indicated a very strong negative (protective) association with BPV infection.

## Discussion

The MHC genes are of particular interest because they are associated with genetic resistance and susceptibility to a wide spectrum of economically important diseases in farm animals (37). It has been suggested that some amino acid motifs forming the pockets in the antigen-binding groove of BoLA-DR molecule are involved in conferring resistance or susceptibility to bovine diseases (20, 27, 38–40). In particular, amino acids within P1, P4, P6, P7, and P9 appear to play a crucial role for determining the degree of immune response (20, 34).

Although downregulation of MHC class I is a property common to BPV E5 oncoprotein (22, 41), no information concerning the potential associations of BoLA class II variability with BPV-2 infection and bladder tumor risk in cattle is available. Therefore, this is the first study that has investigated the relationship between MHC complex class II DRB3 genes, BPV-2 infection status, and bladder tumor risk in cattle.

Our study showed the peptide polymorphic sequences of BoLA-DRB3^*^011:01 and BoLA-DRB3^*^011:02v in several amino acid residues of three pockets. Arg^71^ in P4 was seen in the BoLA-DRB3^*^011:01 allele in healthy animals, whereas Lys^71^ was found in the BoLA-DRB3^*^011:02v allele in diseased cattle. It is conceivable that the amino acid Arg^71^ observed in healthy cattle may be associated with a higher resistance to BPV infection; therefore, these animals may have a lower risk of bladder neoplasia. Indeed RR was indicative of a strong, negative association with BPV infection. Our suggestion is consistent with the results of previous investigations. Indeed it has been shown that Arg^71^ in P4 is correlated with resistance to persistent lymphocytosis caused by the bovine leukemia virus (42). Furthermore, Arg^71^ in P4 has been associated with a high resistance to *Escherichia coli* mastitis in dairy cows (43). Our results appeared to strengthen previous studies suggesting that amino acid residues in P4 are crucial for resistance and/or susceptibility to diseases (34). In addition, Ser^57^ and Asp^57^ were the amino acid residues prevalently found in P9 at position 57 of BoLA-DRB3^*^011:01 and BoLA-DRB3^*^011:02v alleles, respectively. It has been suggested that amino acids encoded at position 57 in P9 of DRB3 alleles play a crucial role in modulating HPV infection in humans, thus contributing significantly to HPV16 infection susceptibility and/or resistance. Indeed it has been hypothesized that Ser^57^ is negatively associated with HPV infection, thus having a protective effect, while Asp^57^ is positively associated to HPV infection, thus contributing significantly to HPV-related cancer risk (44). As in humans, it is conceivable that Ser^57^ may have a protective effect against BPV-2 abortive infection of the urinary bladder of cattle, whereas Asp^57^ of the BoLA-DRB3^*^011:02v allele may contribute to the susceptibility to BPV-2 infection of the urinary bladder. Gln^9^ and Glu^9^ were found in this pocket of BoLA-DRB3^*^011:01 and BoLA-DRB3^*^011:02v alleles, respectively. Gln^9^ may represent a further amino acid motif associated with resistance to virus infection since it has already been shown to play a central role in the resistance to bacterial infection in cattle (43). Furthermore, although this study may indicate a strong positive association between the presence of His^11^ in P6 and the unexpressed BPV *E5* gene, the biological significance of this finding remains to be evaluated. Finally, Asp^45^ was a non-pocket residue found in healthy cattle, replacing Gly^45^ of affected animals, which may corroborate previous studies that suggested that critical immune functions may exist, spanning farther from the binding pockets (45).

Our results are consistent with the suggestion that BoLA class II alleles may influence the immune response to specific BPV-encoded epitopes and, thereby, may play a role in BPV infection that is associated with bladder tumor risk in cattle. Our study indicated that an important association between the DRB3.2^*^22 allele and BPV-2 infection status of the urinary bladder of cattle may exist.

Indeed the sequence analysis of DRB3.2^*^22 allele both in healthy and affected animals revealed two different sequences: DRB3^*^011:01 and DRB3^*^011:02v. DRB3^*^011:01 appeared to have a significant negative association with BPV-2 expression; thereby, DRB3^*^011:01 suggested a protective effect against BPV-2 infection, which may indicate that a lower risk of bladder tumor occurs in those animals carrying this sequence. DRB3^*^011:02v appeared to show a positive association with the viral disease as it was prevalently found in affected animals, and the RR correlated with amino acid residues was 8.1, indicating a strong positive association with BPV infection risk. As in humans (46, 47), it is conceivable that a strong immunity to BPV infection appears to be dependent on MHC class II polymorphism. Indeed it has been suggested that cell-mediated immune responses by T cells bearing CD4 that recognize antigen presented by class II MHC play a crucial role in cancer development by PV infections (48). It is worth noting that the structural analysis of peptides and polymorphism in a comparison study of cattle BoLA-DR and human HLA-DR revealed a very high homology between them (49, 50). Of note is that it has been suggested that the structure and organization of the MHC genes of cattle are very similar to those of the human MHC (34, 51); thereby, ruminant MHC molecules function in a similar manner to human MHC molecules (17).

We realized that similar to previous investigations of the relationships between BoLA DRB3 and microbe infections carried out, including mastitis pathogens such as streptococci, *Escherichia coli*, and *Staphylococcus aureus* (39, 43) and virus infection such as bovine leukemia virus and foot and mouth disease (20, 46, 52, 53), the follow-up necessary to render this study ultimately informative rather than speculative is lacking. On the other hand, the lack of a robust follow-up is believed to be the most important limitation to investigating relationships between cancer risk and HPV infection in human medicine (54). Further studies are needed to better define the role of DRB3^*^ allele polymorphism in BPV infection which is responsible for severe economic losses in the cattle industry and general animal husbandry. Host susceptibility factors and immune responses are very important but poorly understood determinants of persistence and progression of PV infection (55), which warrants further research in this field.

## Data Availability Statement

The raw data supporting the conclusions of this article will be made available by the authors, without undue reservation.

## Author Contributions

SR and ML contributed to the conceptualization. MS, AP, MCa, MCo, and BN contributed to the methodology. ML, VR, and SR contributed to the validation. SR and ML contributed to the writing—review and editing. SR took charge of supervision. All authors contributed to the article and approved the submitted version.

## Conflict of Interest

The authors declare that the research was conducted in the absence of any commercial or financial relationships that could be construed as a potential conflict of interest.
