# Supplementary material for: Association Between BoLA-DRB3.2 Polymorphism and Bovine Papillomavirus Infection for Bladder Tumor Risk in Podolica Cattle
Source: Front Vet Sci. 2021 Jun 9;8:630089. doi: 10.3389/fvets.2021.630089 (PMC8219868; doi:10.3389/fvets.2021.630089)
Supplement: Supplementary file 2 [file Data_Sheet_1.DOCX]

**Supplementary Table S2.** PCR/RFLP, BoLA DRB3.2 allele frequencies in affected, healthy and total sampled animals. Asterisk marks alleles with frequency higher than 5%.

| **PCR/RFLP** |  |  |  |  | **PCR/RFLP** |  |  |  |  | **PCR/RFLP** |  |  |  |
| --- | --- | --- | --- | --- | --- | --- | --- | --- | --- | --- | --- | --- | --- |
| **BoLA DRB3.2*** |  | **Allele**  **Frequence** |  |  | **BoLA DRB3.2*** |  | **Allele**  **Frequence** |  |  | **BoLA DRB3.2*** |  | **Allele**  **Frequence** |  |
| **allele** | **Affected** | **Healthy** | **Total** |  | **allele** | **Affected** | **Healthy** | **Total** |  | **allele** | **Affected** | **Healthy** | **Total** |
| 1 | 0.125 | 0.117 | 0.125 | * | 15 | 0.008 | 0.012 | 0.010 |  | 31 | 0.008 | 0.004 | 0.006 |
| 2 | 0.011 | 0.016 | 0.014 |  | 16 | 0.019 | 0.031 | 0.026 |  | 32 | 0.004 | 0.000 | 0.002 |
| 3 | 0.023 | 0.016 | 0.020 |  | 18 | 0.019 | 0.012 | 0.016 |  | 33 | 0.019 | 0.016 | 0.018 |
| 5 | 0.004 | 0.000 | 0.002 |  | 20 | 0.008 | 0.020 | 0.014 |  | 34 | 0.000 | 0.004 | 0.002 |
| 6 | 0.008 | 0.012 | 0.010 |  | 21 | 0.027 | 0.004 | 0.016 |  | 35 | 0.004 | 0.000 | 0.002 |
| 7 | 0.053 | 0.094 | 0.074 | * | 22 | 0.057 | 0.121 | 0.089 | * | 36 | 0.004 | 0.000 | 0.002 |
| 8 | 0.053 | 0.063 | 0.058 | * | 23 | 0.011 | 0.016 | 0.014 |  | 37 | 0.000 | 0.004 | 0.002 |
| 9 | 0.019 | 0.004 | 0.012 |  | 24 | 0.023 | 0.008 | 0.016 |  | 41 | 0.015 | 0.055 | 0.035 |
| 10 | 0.080 | 0.047 | 0.064 | * | 26 | 0.042 | 0.020 | 0.031 |  | 42 | 0.030 | 0.008 | 0.019 |
| 11 | 0.053 | 0.074 | 0.064 | * | 27 | 0.087 | 0.043 | 0.066 | * | 44 | 0.004 | 0.000 | 0.002 |
| 12 | 0.038 | 0.043 | 0.041 |  | 28 | 0.091 | 0.090 | 0.091 | * | 51 | 0.011 | 0.012 | 0.012 |
| 13 | 0.004 | 0.016 | 0.010 |  | 29 | 0.008 | 0.016 | 0.012 |  |  |  |  |  |
| 14 | 0.000 | 0.004 | 0.002 |  | 30 | 0.000 | 0.004 | 0.002 |  |  |  |  |  |

|  |
| --- |
