# Supplementary material for: Association Between BoLA-DRB3.2 Polymorphism and Bovine Papillomavirus Infection for Bladder Tumor Risk in Podolica Cattle
Source: Front Vet Sci. 2021 Jun 9;8:630089. doi: 10.3389/fvets.2021.630089 (PMC8219868; doi:10.3389/fvets.2021.630089)
Supplement: Supplementary file 3 [file Data_Sheet_2.DOCX]

**Supplementary Table S3**

Amino acid sequences of BoLA-DRB3.2 binding groove region. From the left: Subject Status: “A”= Affected; “H”= healthy; Subject; “RFLP” = PCR/RFLP DRB3.2*22 allele single (-het) or double (-hom) dose; Sequence allele name (IPD-MHC web site); Amino acid sequence alignment to BoLA09801|BoLA-DRB3*011:01, consistency is reported as *; pocket (1,4,6,7,9) and residue position number (9, 11, 13, 28, 30, 37, 47, 57, 60, 61, 67, 70, 71, 74, 78, 82, 86, 89) are in accordance with Sharif et al., (2000).

**Pocket 9 6 4 7 7 9 7 9 97 7 44 4 4 1 1 1**

**Residue 9 1 1 2 3 3 4 5 66 6 77 7 7 8 8 8**

**1 3 8 0 7 7 7 01 7 01 4 8 2 6 9**

**Sbj. Allele**

**S. n. RFLP Sequence**

A 165 *22-het BoLA-DRB3*11:01 QYHKGECHFF NGTERVRLLD RHFYNGEEYV RFDSDWDEFR AVTELGRPSA EYWNSQKDFL ERRRAEVDTV CRHNYGVVES FTV

A 262 *22-het BoLA-DRB3*11:01 ********** ********** ********** ********** ********** ********** ********** ********** ***

A 004 *22-het BoLA-DRB3*11:02v E*Y******* ********** ********** ******G*** ********D* ********** **K******* ********** ***

A 006 *22-het BoLA-DRB3*11:02v E*Y******* ********** ********** ******G*** ********D* ********** **K******* ********** ***

A 007 *22-het BoLA-DRB3*11:02v E*Y******* ********** ********** ******G*** ********D* ********** **K******* ********** ***

A 247 *22-het BoLA-DRB3*11:02v E*Y******* ********** ********** ******G*** ********D* ********** **K******* ********** ***

A 252 *22-het BoLA-DRB3*11:02v E*Y******* ********** ********** ******G*** ********D* ********** **K******* ********** ***

A 255 *22-het BoLA-DRB3*11:02v E*Y******* ********** ********** ******G*** ********D* ********** **K******* ********** ***

A 265 *22-het BoLA-DRB3*11:02v E*Y******* ********** ********** ******G*** ********D* ********** **K******* ********** ***

A 267 *22-het BoLA-DRB3*11:02v E*Y******* ********** ********** ******G*** ********D* ********** **K******* ********** ***

H 033 *22-hom BoLA-DRB3*11:01 ********** ********** ********** ********** ********** ********** ********** ********** ***

H 135 *22-hom BoLA-DRB3*11:01 ********** ********** ********** ********** ********** ********** ********** ********** ***

H 143 *22-hom BoLA-DRB3*11:01 ********** ********** ********** ********** ********** ********** ********** ********** ***

H 146 *22-het BoLA-DRB3*11:01 ********** ********** ********** ********** ********** ********** ********** ********** ***

H 222 *22-het BoLA-DRB3*11:01 ********** ********** ********** ********** ********** ********** ********** ********** ***

H 231 *22-het BoLA-DRB3*11:01 ********** ********** ********** ********** ********** ********** ********** ********** ***

H 223 *22-het BoLA-DRB3*11:02v E*Y******* ********** ********** ******G*** ********D* ********** **K******* ********** ***

H 226 *22-het BoLA-DRB3*11:02v E*Y******* ********** ********** ******G*** ********D* ********** **K******* ********** ***

H 302 *22-het BoLA-DRB3*11:02v E*Y******* ********** ********** ******G*** ********D* ********** **K******* ********** ***
